# Bibliography

**Published:** 1893-03

**Authors:** 


### Bibliography.

A STUDY OF THE DEGENERACY OF THE JAWS OF THE HUMAN RACE. By Eugene S. Talbot, M.D., D.D.S., Chicago, Illinois. Published by the S. S. White Dental Manufacturing Company Philadelphia, 1892.

This book, though comparatively limited in size,—sixty-seven pages,—embodies much thought and critical examination of cases by the author, as well as many others interested in similar studies. It would be unjust to criticise such a work as this by the ordinary methods. Very few have had the experience of the author in the direction in which he has made his life-work, and, therefore, while some of his conclusions may be at variance with individual opinions, it may be well to remember that they are not based on speculation but on careful, painstaking observations of many years. The adverse criticisms with which this book has been met since its issue has led the present writer to infer that it has crushed some pet theories. This should make it more valuable, for a book that fails to do this lacks the necessary life to justify an existence.

Whatever opinions may be held adverse to his deductions, it seems certain that no one can rise from the reading without having the mind broadened by suggestive thought.

The author's conclusions in the opening pages are not very encouraging for the human race, for it would seem, if he be correct, that in proportion to the extent of intellectual advancement will the race deteriorate, for he says, "Children born of neurotic parents are apt to inherit brilliant minds and very weak constitutions. They are very bright scholars, always standing at the heads of their classes. . . . If they grow up they frequently become geniuses or insane, drunkards, criminals, or egotists." This idea is repeated in the latter part under the head of neurosis, in which he says, "It is a singular fact that there seems to be a fascination for neurotic individuals to seek one another's society. Hence the large number of literary societies which are being formed throughout the country, entertainments, and social gatherings, at which brilliant men and women are to be found." All this is very discouraging if accepted. That it is true we have no doubt, for intelligence must always prefer intelligence to stupidity ; but that it leads to the dire results pictured cannot be accepted without qualification. If it be true, there is and always must be but one result,—development from barbarism to a higher cultivation, and, the limit reached in a special line, there is either extinction or a return to barbarism. It is a very discouraging view to take of life, and yet it is to be feared it has stubborn facts to support it.

After quoting many writers to show that the jaws are degenerating, he says, "Actual measurement supports this theory. . . . Evolutionists and scientists have frequently mentioned the fact that the jaws are changing in shape and size ; but they have never produced data just to show what changes have taken place. I have, therefore, collected a large number of measurements for the purpose of obtaining facts in regard to these changes. The measurements of the skulls of the early races, as well as modern people, were made from specimens in the museums and crypts of churches in Europe.

Then follows a list of persons in different countries, and sections of this country, who have laboriously aided to make the tables which follow satisfactory by their completeness.

It is to be regretted that, owing to ill health, the author was not able to give careful attention to the proof. There have been some errors in consequence, and it has been requested that in any notice given attention should be called to these typographical errors. "On page 22, where the word Chicago is used, 51 M. and 51 F. should be written. At the bottom of page 18 the number of antero-posterior measurements made by me of seven hundred and twelve male and female mouths of individuals and models are as follows:

| | Lowest. | Highest. | Average. |
|---|---|---|---|
| Inches | 1.56 | 2.19 | 1.84 |
| Mille | 39.69 | 55.56 | 46.74 |

It is necessary to group both male and female, because many of the measurements are taken from plaster casts, so that I was unable to determine the sex."

The temptation is to follow the direction of the author's thought still farther, but space will not permit attention to more than one idea somewhat extensively elaborated in the latter pages. The statement is, that "If all the people of the world lived in cities, the human race would become extinct in two and one-half centuries." If by this is meant, and the text indicates it, that the active life of cities tends to destruction of the race, then there must be a difference of opinion. If "all men lived in cities" life would become impossible, for the soil must be made to bring forth food necessary, and men must be ready to meet this want. The idea, however, that city life, properly conducted, is destructive, is a fallacious one, and is not borne out, as far as the writer is aware, by statistics.

The dental profession and the scientific world at large owe much to Dr. Talbot and his collaborators; but we fear the reward they will receive will not come in this generation, for the average reader is not fond of details of measurement, and will persist in regarding them as very uninteresting reading. This character of work, however, can have no ephemeral life, but will become the basis for more advanced conclusions generations to come.

The Rise, Fall, and Revival of Dental Prosthesis. By B. J. Cigrand, B.S., D.D.S., Chicago, Illinois.

This claims to be a history of mechanical dentistry, but it can hardly be said to justify the title, or to present the subject in a way that will prove satisfactory to the student desirous of obtaining the most accurate information.

While it claims to be a compilation of facts, the entire absence of sources of information leads to the suspicion of inaccuracy, and conveys the impression that the author has not carefully tested the truth of his statements.

Notwithstanding this defect, the book is a very readable one, the author having succeeded in investing the subject with interest.

It is extremely unfortunate that, with his apparent care in illustrations and general excellent taste in press-work and paper, he should have neglected careful proof-reading. It has rarely been our privilege to see so much inexcusable carelessness in this direction.

NOTE-BOOK FOR DENTAL STUDENTS. (Dental Anatomy and Physiology.) By James F. Rymer, Member of the Royal College of Surgeons of England; Licentiate in Dental Surgery of the Royal College of Surgeons of England; Doctor of Dental Surgery, University of Pennsylvania. Second edition. London: Claudius Ash & Sons, Limited, 1892.

This note-book of sixty pages was issued in the first edition in 1888, as the author states, "with the express object of proving useful prior to examinations. . . . Accordingly, I have collected together the chief matter connected with dental anatomy and physiology." He further very properly says, "To understand so large a subject in this condensed form, it is necessary that the general text-books should be first mastered."

The notes cover the "Teeth of Man," with their peculiar characteristics; "The Eruption of Teeth;" definition of "Terms as Applied to Types;" "Dental Tissues" and adjacent parts, and the "Development of Teeth." These subjects are succeeded by very compact but clearly expressed notes on "Comparative Dental Anatomy."

The writer knows of no book of its size that comprises so much condensed information. As an aid to memory for those for whom it is specially prepared, the book can be recommended without reserve, and it will also be found a very convenient pocket reference for those of more enlarged experience.

